# Impact of the Timing of Umbilical Cord Clamping on Maternal and Neonatal Outcomes in Saudi Arabia

**DOI:** 10.7759/cureus.53536

**Published:** 2024-02-04

**Authors:** Bayan Sonbol, Abeer Orabi, Hend Al Najjar

**Affiliations:** 1 Obstetrics and Gynecology, King Salman bin Abdulaziz Medical City, Madinah, SAU; 2 College of Nursing, King Saud bin Abdulaziz University for Health Sciences, Jeddah, SAU; 3 Nursing, King Abdullah International Medical Research Center, Jeddah, SAU

**Keywords:** saudi arabia, birth practices, neonatal outcomes, maternal outcomes, umbilical cord clamping, timing of cord clamping, delayed cord clamping, early cord clamping

## Abstract

Introduction: The optimal time for umbilical cord clamping after delivery has been under debate for several decades. This study aimed to assess the time-dependent effects of umbilical cord clamping on maternal and neonatal outcomes.

Methods: An observational correlational design was used to recruit 161 pregnant women conveniently. Outcomes were observed and recorded using a structured checklist developed by the authors. Pregnant females aged ≥18 years, with uncomplicated delivery, and who were willing to participate were recruited. Exclusion criteria included stillbirths, newborns with congenital anomalies, newborns too small for their gestational age, intra-uterine growth restriction, nuchal cord, and meconium-stained liquor.

Results: The mean age of the participants was 29.93 ± 6 years. Early clamping (<1 minute) was performed for 93.8% of the participants with a mean of 29.58 ± 18 seconds. Delayed clamping was associated with a decrease in blood loss and the length of hospital stay in addition to an increase in first-minute APGAR score and neonatal temperature (P < 0.05).

Conclusions: Delayed cord clamping was associated with improved maternal and neonatal outcomes.

## Introduction

The timing of umbilical cord clamping (UCC) during childbirth varies depending on the clinical policy and practice [1]. It has historically been immediate, aiming for a rapid conclusion of the birthing process. However, emerging evidence over the past decades has challenged this approach [2]. Delayed cord clamping (DCC), generally defined as clamping the umbilical cord at least one minute after birth, allows for continued blood flow from the placenta to the newborn, aiding the neonate's cardiovascular transition and reducing the risk of iron deficiency [3].

Recent systematic reviews and meta-analyses highlight the significance of DCC in reducing the risk of neonatal mortality, especially for premature babies [4]. These advantages align with recommendations from the World Health Organization and the National Institute for Health and Care Excellence that advocate for DCC to enhance labor outcomes [5,6]. Conversely, early cord clamping (ECC) (<1 minute) potentially deprives the term newborn of 20-30 mg/kg of iron, which would otherwise satisfy the neonatal needs for three months [7].

DCC is associated with numerous neonatal benefits, including higher hemoglobin levels, increased iron storage, improved neurodevelopment, reduced need for blood transfusions, and lower rates of chronic diseases in preterm infants [4]. In term infants, DCC increases hemoglobin levels at birth and improves iron stores in the first months of life [8]. Moreover, placing the newborn below the level of the placenta has been shown to significantly increase hemoglobin and hematocrit at 3-4 months with no adverse consequences [9-11]. 

The implications of UCC timing extend beyond neonatal health, influencing maternal outcomes, such as postpartum hemorrhage (PPH) and the duration of the third stage of labor [12]. Studies reported that DCC is not associated with an increased risk of PPH or the need for maternal blood transfusion, regardless of the delivery method [4,13]. Furthermore, the study by Kuo et al. evaluated maternal outcomes after DCC with singleton and twin pregnancies and found no increase in the risk of PPH [14]. Moreover, the study by Ruangkit et al. found no significant difference in the rate of PPH following DCC performed for mothers with multiple pregnancies [15]. Furthermore, DCC did not increase the risk of retained placenta or maternal blood loss.

Potential adverse effects, such as increased incidence of pathological jaundice, have been reported with DCC [16]. However, a cross-sectional study verified no association between the timing of UCC and pathological jaundice in low-risk neonates [17].

The timing of UCC during childbirth has been a subject of considerable debate in obstetric and neonatal health fields. Despite accumulating evidence supporting DCC, controversies and research gaps persist. The optimal time for UCC remains a significant question to reduce risks and improve outcomes [18]. However, limited studies have been conducted in Saudi Arabia to assess the effect of UCC timing on maternal and neonatal outcomes. This paper seeks to contribute to the ongoing research on UCC timing, aiming to delineate its impact on both maternal and neonatal outcomes and to provide clarity and direction for future research and clinical guidelines in this vital aspect of perinatal care.

## Materials and methods

Methodology

Study Design and Setting

An observational correlational design was used to conduct this study in the labor and delivery unit at King Abdulaziz Medical City.

Sample Size

The sample size was determined using G*Power online software. We aimed to include 145 participants to achieve an 85% power level, with 0.5 medium effect size, 5% error probability, and 10% estimated missing data. The final sample size was 161.

Study Subjects

Pregnant females aged ≥18 years, with uncomplicated delivery, and who were willing to participate were recruited. Exclusion criteria included stillbirths, newborns with congenital anomalies, newborns too small for their gestational age, intra-uterine growth restriction, nuchal cord, and meconium-stained liquor.

Ethical Considerations

Approval to conduct the study was granted by the Institutional Review Board (IRB) at King Abdulaziz Medical City-Western Region Hospital (SP19/090/J). The researchers explained the study's aim to eligible females and obtained their informed consent. Confidentiality, privacy, and anonymity of participants were rigorously maintained.

Data Collection

A structured checklist developed after an extensive literature review [17,19-21] was used. The checklist comprised five sections: sociodemographic characteristics; obstetrical history; labor data; maternal outcomes, such as the duration of the third stage of labor, estimated blood loss (mL), early PPH, and length of hospital stay; and neonatal outcomes, encompassing cord hemoglobin level, APGAR score at the first and fifth minutes after delivery, pulse and temperature, SpO_2_, admission to neonatal intensive care unit (NICU), pathological jaundice, respiratory distress, and neonatal death. The checklist's validity was confirmed by experts in the field and the reliability was ensured through pilot testing on 20% of the sample by a qualified observer. Data were collected over a period of four months. Each participant’s sociodemographic data and obstetrical history were collected using interviews. Then, each participant was observed during the second stage of labor up to 24 hours post-delivery using checklists and stopwatches for cord clamping timing. The necessary data were recorded from the electronic medical record system.

Data Management and Analysis

Data were analyzed using IBM SPSS Statistics for Windows, Version 28 (Released 2021; IBM Corp., Armonk, New York, United States) [22]. Data were summarized using the arithmetic means, standard deviations, frequencies, and percentages. Pearson correlation and regression analyses were used to examine the associations between the study variables and identify outcome predictors. A P-value ≤ 0.05 indicated significant results between examined variables.

## Results

The participants ranged in age from 18 to 42 years, with a mean of 29.93 ± 6 years. Half of the participants had a high school education (n = 73, 45.4%). The majority of the participants were unemployed (n = 141, 87.6%) and Saudi (n = 158, 98.1%) (Table 1).

**Table 1:** Sociodemographic Characteristics SD, standard deviation

Variable	Mean ± SD	
Age (N = 161)	29.93 ± 6.02	
Variables	Frequency (N = 161)	%
Education Level		
Primary school	7	4.3
Intermediate school	13	8.1
High school	73	45.4
Bachelor’s degree or higher	68	42.2
Occupation		
Employed	20	12.4
Unemployed	141	87.6
Nationality		
Saudi	158	98.1
Non-Saudi	3	1.9

The gestational age ranged between 34 and 41 weeks, with a mean of 38.8 ± 1 weeks. Three-quarters of the participants (n = 122, 75.78%) were multipara, (n = 80, 65.7%) of them had a spontaneous vaginal delivery, and (n = 113, 92.7%) had no complications in their previous delivery (Table 2).

**Table 2:** Obstetrical History PPH, postpartum hemorrhage

Variables	Frequency (N = 161)	%
Nullipara	39	24.22
Multipara	122	75.78
Previous Mode of Delivery (N = 122)		
Spontaneous vaginal delivery	80	65.7
Assisted vaginal delivery	3	2.4
Caesarean section	39	31.9
Previous Complications (N = 122)		
No	113	92.7
PPH	2	1.7
Shoulder dystocia	1	0.8
Cord presentation	3	2.4
Abnormal foetal presentation	3	2.4

Fetal presentation was cephalic for (n = 153, 95%), cardiotocograph (CTG) interpretation was reassuring for (n = 144, 89.4%), and the mode of delivery was vaginal for (n = 115, 71.4%) of the participants. The majority of the participants (n = 151, 93.8%) had ECC with a mean time of 29.58 ± 18.37 seconds (Table 3).

**Table 3:** Labor Data CTG, cardiotocograph; UCC, umbilical cord clamping

Variables	Frequency (N = 161)	%
Fetal Presentation		
Cephalic	153	95
Breech	8	5
CTG Interpretation		
Reassuring	144	89.4
Non-reassuring	17	10.6
Mode of Delivery		
Vaginal delivery	115	71.4
Caesarean section	46	28.6
Timing of UCC		
Early	151	93.8
Delayed	10	6.2

The mean duration of the third stage of labor and the length of hospital stay were 5.087 ± 4 minutes and 2.5 ± 9 days, respectively. Less than half of the participants (n = 64, 39.8%) lost 100-200 mL of blood. Only (n = 3, 1.9%) of the participants had early PPH. Concerning the neonatal outcomes, the means of cord hemoglobin level, first-minute and fifth-minute APGAR scores, pulse rate, temperature, and SpO_2_ were 15.4 ± 1.5, 8.8 ± 0.5, 8.9 ± 0.7, 147.4 ± 10.5, 36.6 ± 0.18, and 96.9 ± 1.9, respectively. The majority of the newborns did not have pathological jaundice (n = 155, 96.3%), respiratory distress (n = 154, 95.7%), or were admitted to the NICU (n = 134, 83.2%). No neonatal deaths occurred among the newborns (Table 4).

**Table 4:** Maternal and Neonatal Outcomes PPH, postpartum hemorrhage; NICU, neonatal intensive care unit

Variables	Frequency (N = 161)	%
Estimated blood loss (mL)		
100–200	64	39.8
300–400	48	29.8
More than 500	49	30.4
Early PPH		
Yes	3	1.9
No	158	98.1
Admission to the NICU		
Yes	27	16.8
No	134	83.2
Pathological Jaundice		
Yes	6	3.7
No	155	96.3
Respiratory Distress		
Yes	7	4.3
No	154	95.7

There were no significant associations between the timing of UCC and the duration of the third stage of labor (R = 0.115, P = 0.146) or early PPH (R = 1.031, P = 0.491) among the participants. Multinomial logistic regression showed that for each second delay in UCC, 100-200 mL and 300-400 mL estimated blood loss were more likely to increase compared to >500 mL, with a high statistical significance at 95% confidence (R = 1.058, P = 0.001) and (R = 1.059, P = 0.001), respectively. Finally, the data revealed a statistically significant negative association between the timing of UCC and the length of hospital stay (R = 0.230, P = 0.003). With regard to neonatal outcomes, no significant associations were found between the timing of UCC and cord hemoglobin level (R = 0.055, P = 0.490), SpO2% (R = 0.035, P = 0.661), pathological jaundice (R = 1.035, P = 0.289), and respiratory distress (R = 1.049, P = 0.159). However, significant correlations were found between the first-minute APGAR score and the timing of UCC (R = 0.184, P = 0.020), pulse rate (R = -0.161, P = 0.041), and temperature (R = 0.474, P = 0.001.). Furthermore, each second delay in the UCC reduced the probability of admission to the NICU by 1.060 times (P = 0.002) (Table 5).

**Table 5:** Association Between the Timing of UCC and Maternal and Neonatal Outcomes UCC, umbilical cord clamping; NICU, neonatal intensive care unit; *indicates significance

Variable		R	P-value
Estimated blood loss in mL	Category (100–200)	1.058	0.001*
	Category (300–400)	1.059	0.001*
Length of hospital stay		-0.230	0.003*
First-minute Apgar score		0.184	0.02*
Pulse		-0.161	0.041*
Temperature		0.474	0.001*
Admission to NICU		1.060	0.002*

## Discussion

This study showed no significant association between the timing of UCC and the duration of the third stage of labor. However, for each second delay in UCC, the likelihood of decreasing estimated blood loss rises, thus improving maternal outcomes. These findings align with the finding from the study by Nelin et al. [11], who found that DCC was not associated with the increased length of the third stage of labor or increased maternal blood loss in a tertiary hospital in Nepal. Moreover, in a randomized controlled trial comparing maternal blood loss with ECC versus DCC in females undergoing cesarean section at term, DCC was not associated with increased maternal blood loss [23]. Furthermore, no significant association was found between the timing of UCC and early PPH in multiple pregnancies [15].

The present study found no significant relationship between UCC timing and the cord hemoglobin level. However, there was a significant relationship between the first-minute APGAR score and UCC timing. This association contrasts with a previous study that indicated that DCC increased neonatal hemoglobin levels [3]. This discrepancy might be due to variations in the timing of UCC, differences in sample sizes, or methodologies employed. However, the study aligns with the study by Lagatta et al. [10], suggesting that DCC positively affects neonatal outcomes, including APGAR scores, by ensuring better blood circulation and oxygenation at birth.

This study associates DCC with slower, yet normal, pulse rates in neonates, suggesting that DCC increases neonatal blood volume and stabilizes the pulse rate. However, this finding contrasts with a study that investigated physiologically based cord clamping versus ECC and found no significant difference in mean heart rates between the two groups [24]. These variations emphasize the complexity of neonatal responses to UCC timing under different clinical scenarios and infant populations.

The present study indicated that DCC was significantly associated with increased neonatal temperature. This result contradicts the results obtained by Jain et al. [20], who found no significant difference in temperature between pre-term and term infants who underwent DCC. These results suggest that the benefits of DCC outweigh its risks. Furthermore, there was no significant relationship between UCC timing, and the incidence of pathological jaundice, and similar results were reported in low-risk newborns [17]. 

Study limitations

The use of a small sample size and convenience sampling may limit the generalizability of the findings to the broader population.

Recommendations

Implementation of DCC practice in healthcare settings alongside educational programs for healthcare providers about the advantages of DCC is recommended.

## Conclusions

The results of this study indicate that DCC was not associated with harmful effects; nevertheless, it seems to improve maternal and neonatal outcomes. DCC was associated with a decrease in estimated blood loss and a shorter length of hospital stay. Concerning neonatal outcomes, DCC tends to positively affect the first-minute Apgar score, increase body temperature, and decrease the pulse rate. Finally, each second delay in the timing of UCC was associated with a reduced probability of newborn admission to the NICU. In conclusion, the study suggests that the timing of UCC can significantly impact population health, emphasizing the importance of identifying optimal timepoints and approaches for managing childbirth.
